# Health risk assessment of heavy metals in imported frozen bovine meat and organs marketed in Sohag, Egypt

**DOI:** 10.1038/s41598-025-29927-x

**Published:** 2025-12-14

**Authors:** Mohammed Ashry Rabeey, Rana Fahmi Sabala, Amira Ibrahim Zakaria, Khalid Ibrahim Sallam

**Affiliations:** Food Hygiene, Safety, and Technology Department, Faculty of Veterinary Medicine, Mansoura University, Mansoura, 35516 Egypt

**Keywords:** Heavy metals, Beef, Liver, Health risk assessment, Mercury, Lead, Cadmium, Biochemistry, Environmental sciences, Risk factors

## Abstract

Heavy metals like lead, cadmium, and mercury can accumulate in meat and organ tissues, posing significant food safety and health risks. In the current study, 315 frozen imported bovine samples (105 each of muscle, liver, and kidney) collected from local markets in Sohag governorate, Egypt, were analyzed by atomic absorption spectrophotometry for the determination of mercury (Hg), lead (Pb), and cadmium (Cd) concentrations. The metals determined were investigated for their potential public health hazards in humans. The mean ± SE concentrations of Hg, Pb, and Cd in beef were 0.312 ± 0.058, 0.684 ± 0.105, and 0.030 ± 0.005, respectively, while the corresponding values in liver samples were 0.273 ± 0.054, 0.763 ± 0.106, and 0.056 ± 0.007, respectively, and in kidney samples were 0.167 ± 0.04, 0.716 ± 0.119, and 0.073 ± 0.014, respectively. Beef samples contained the highest Hg level, while liver samples contained the highest level of Pb, and kidney samples contained the highest level of Cd. More than half of the samples tested exceeded the permissible limits set by International and local food agencies for Hg and Pb; exactly, 62.9, 60, and 54.3% of muscle, liver, and kidney samples, respectively, exceeded the maximum limits of Pb, while 54.3 and 57.1% of muscle and liver samples exceeded the limit of Hg. In contrast, none of the tested liver or kidney samples exceeded the limit of Cd, although only 22.9% of the beef samples exceeded their maximal limit. Although the human health risks estimated in the current study for consuming the contaminated samples were low, high proportions of samples exceeded the permissible limit, and therefore, continuous monitoring of heavy metal residues in food, especially of animal origin, is of great significance.

**Supplementary Information:**

The online version contains supplementary material available at 10.1038/s41598-025-29927-x.

## Introduction

Beef is considered one of the most important sources of protein, besides the essential nutrients, including fats, vitamins, and minerals^1^. In addition, beef has a high proportion of unsaturated fatty acids in addition to being a source of conjugated linoleic acid, which acts on human health as an anti-inflammatory, antithrombotic, and atherosclerotic preventive^2^. Therefore, the consumption of beef has increased largely worldwide, and it is becoming a preference among consumers, particularly in the Middle East, due to its unique nutritional values with low content of intramuscular fat, saturated fatty acids, and cholesterol when compared to other red meats^3,4^.

Due to rising demand for beef as a source of animal protein, along with the population increasing to 108 million by 2025 and limited domestic production, Egypt imports frozen beef from Brazil, India, and the USA to meet this need.

Bovine samples, including beef, liver, and kidney, are easily contaminated by different sources of hazards. Heavy metals can contaminate animal tissues through many entry routes, such as grazing behavior in cattle in contaminated soils, which leads to the accumulation of heavy metals in their body via ingestion^5^. Also, animal feed and water are contaminated by the surrounding environment and constitute an additional source of contamination^6^. Contamination with heavy metals is a serious threat because of their toxicity, bioaccumulation, and biomagnification in the food chain^7^. These pollutants often have direct physiological toxic effects because they are stored or incorporated in tissues, sometimes permanently^8^. Heavy metals can interfere with the functions of enzymes and are responsible for many diseases, especially cardiovascular, renal, and even bone disorders. Some metals are known to be carcinogenic, mutagenic, and teratogenic in experimental animals^9^. Lead is recognized as a neurotoxic agent, which can impair cognitive performance in children and contribute to cardiovascular diseases, increasing blood pressure in adults^10^. Cadmium is associated with various carcinogenic conditions^11^. Mercury exists in nature in different forms, including metallic and inorganic mercury. Acute exposure to elemental mercury vapor can lead to fatal pneumonitis^12^.

The consumption of toxic metal-contaminated meat over an extended period may cause toxicity; therefore, the main objective of this work is to evaluate mercury, lead, and cadmium concentrations in imported frozen bovine meat, liver, and kidney distributed in Sohag local market, Egypt, as well as to assess the possible hazards of consuming such bovine tissues on the health of the Egyptian general population through calculating the estimated daily intake (EDI), Target Hazard Quotients (THQ), Hazard Index (HI), and Cancer risk (CR).

## Materials and methods

### Sample collection

A total of 315 imported frozen bovine samples comprising meat, liver, and kidney samples (105 each) were randomly collected from local markets in Sohag City, Egypt. Each sample was separately packed in a clean polyethylene bag, labeled with the sample tissue type and number, and kept frozen until transferred to the Laboratory of Food Hygiene, Safety, and Technology, College of Veterinary Medicine, Mansoura University, for digestion and analysis for heavy metal determination.

### Preparation and digestion of samples

**Fig. 1:**
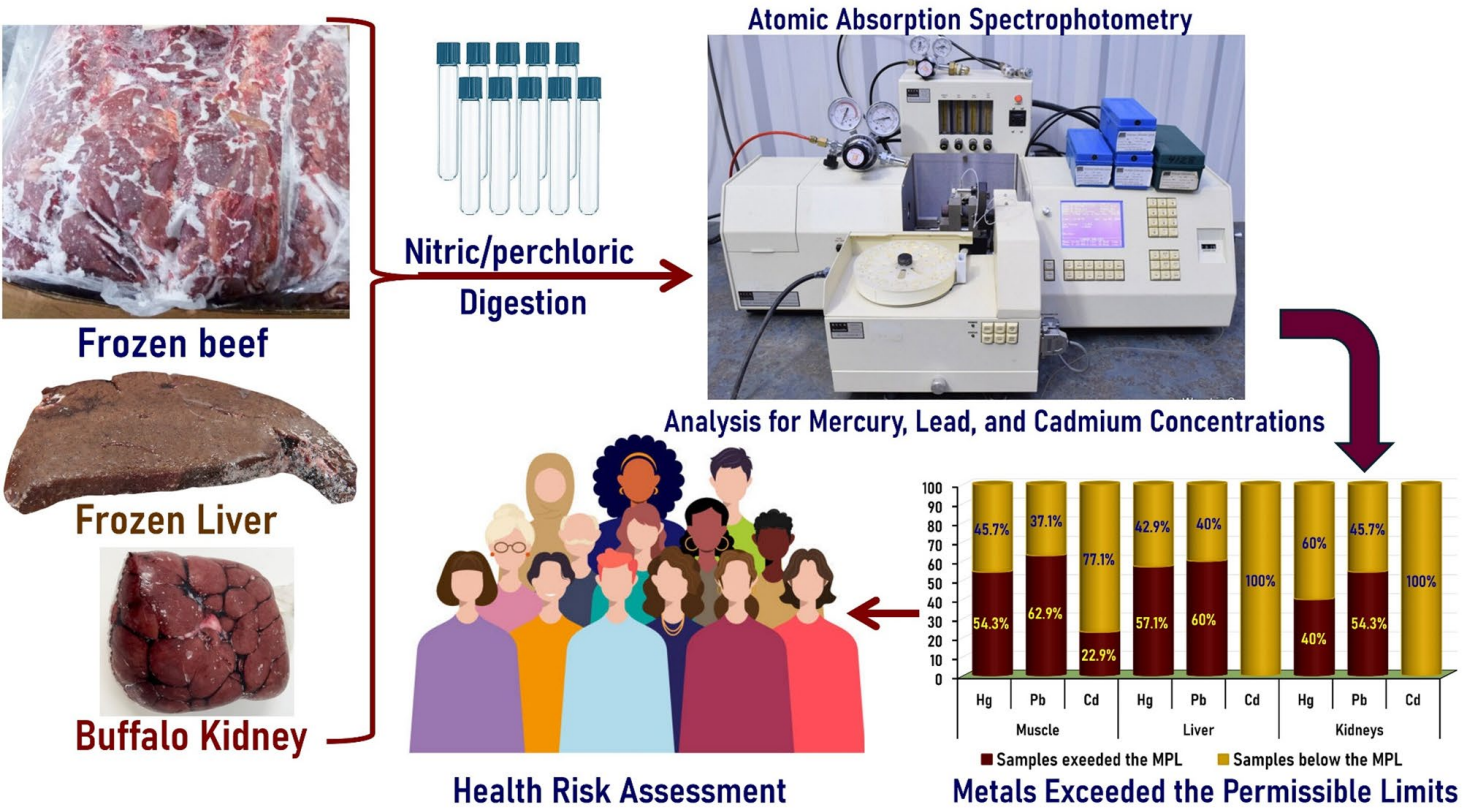
Graphical summary of the study design.

Nitric acid (HNO_3_, ACS reagent ≥ 90.0%; Molecular Weight: 63.01) and Perchloric acid (HClO_4_, ACS reagent 70%; Molecular Weight: 100.46) were obtained from Merck (Merck KGaA, Darmstadt, Germany). For sample digestion and analysis, 2 g of each sample were taken and dissected using a stainless steel scalpel and forceps, then macerated into small pieces and homogenized in a 20 mL screw-capped tube, containing a mixture of 12 mL concentrated nitric-perchloric acid (2:1), and placed overnight in a water bath adjusted at 55 °C for complete digestion of the samples. The digested samples were cooled to room temperature, diluted with deionized water, and then filtered through Whatman Filter Paper Grade 42 (Merck KGaA, Darmstadt, Germany) into a clean glass beaker. Subsequently, the filtrate was made up to 50 mL with deionized water and analyzed for the selected heavy metals (mercury, lead, and cadmium). Blank solutions of the reagents were digested by the same technique, but without samples to define the background correction of the reagents. The study design chart is shown in Fig. 1.

### Heavy metals analysis

All aliquots of the samples sent to be analyzed at the Central Laboratory, Faculty of Veterinary Medicine, Zagazig University, Egypt, for the metals; Hg, Pb, and Cd using “Buck Scientific USA 210 VGP Atomic Absorption Spectrophotometer” provided with an oxidizing air acetylene flame for analysis of Pb and Cd, while mercury hydride system (MHS) “cold vapor technique” was used for determination of Hg. Spectral lamps with wavelengths of 253.65 nm, 283.31 nm, and 228.80 nm were adopted for mercury, lead, and cadmium, respectively. The concentration of metals (mg/kg wet weight) was calculated using the following equation:

$${\text{Element}}={\text{R}} \times {\text{D/W}}$$

where R is the reading of the element concentration (mg/kg) from the digital scale of AAS, D is the final volume of the prepared sample (mL), and W is the wet weight of the sample (g).

### Validation of analytical methods

Method validation has been proven as previously described by Sabala et al.^13^. To create the standard solutions used for the calibration curves, 1000 mg/L of each metal was diluted with acidified ultrapure water (5% v/v HNO3) in accordance with the suggested AOAC Official Method 2015.01. Standard solutions of 0.0, 0.01, 0.05, 0.1, 0.5, 2, and 5 µg/L were used for Hg; 0.0, 0.01, 0.02, 0.1, 0.5, 5, and 10 µg/L were used for Pb; 0.0, 0.005, 0.01, 0.05, 0.25, 2.5, and 5 µg/L were used for Cd. The correlation coefficient (R2) calculated for all calibration curves of the metals analyzed was 0.999.

In order to ensure the accuracy of the results, the analytical method was validated by measuring the linearity of the calibration curve, the detection limit, the quantification limit, the recovery percentage of the certified reference material, and the spiking recoveries of all heavy metal analytes. For the instrumental precision, LOD and LOQ were determined based on the calibration curve as follows:

$${\text{LOD}}/{\text{LOQ}}={\text{F}} \times {\text{SD}}/{\text{b}}$$

Where - F: Factor of 3.3 for LOD and 10 for LOQ; SD: Standard deviation of the blank; b: Slope of the regression line. The mean of spike recovery (%) is calculated by dividing the mean of the recovered amount by the mean of the spiked amount. To ensure the accuracy of the results, five consecutive measurements were made for the metals studied in the certified reference material (dogfish liver DOLT-4).

In order to estimate the precision of the digestion method, known concentrations of the metals under analysis were added to the fish tissues. The digestion and analysis were then carried out using the same analytical technique used to measure the metals in the fish samples. Recovery rates of 96.4%, 98.9%, and 95.9%, based on the concentrations of the spiked amount, were calculated for Hg, Pb, and Cd, respectively.

### Health risk assessment associated with the consumption of contaminated beef and liver samples

#### Estimated daily intake

The estimated daily intake (EDI) of heavy metals (µg per day per person) was calculated by multiplying the mean concentration of the metals detected in beef or liver bovine tissue samples by the average daily consumable rates of such samples. The calculation of the EDI of tested heavy metals was done using the following formula established by AOAC^14^.

$${\text{EDI}}={\text{MC}} \times {\text{IR}}$$

MC is the metal concentration (µg/g) in the examined bovine tissues (meat and liver) on a wet weight basis. IR is the average daily ingestion rate of imported bovine meat (7.1 g/day or 0.0071 kg/day) and liver (2.39 g/day or 0.00239 kg/day) for a 70 kg adult Egyptian consumer, calculated according to USDA (2022)^15^ and USDA (2020)^16^, respectively.

#### Target hazard quotient (THQ)

THQ is applied to assess the probable non-carcinogenic health hazards related to heavy metal exposure via adult consumption of imported frozen beef, liver, and kidney samples over a lifetime. The hazard quotient is calculated using the equation established by Chien et al. (2002)^17^.

$${\text{THQ}}={\text{EDI}}/{\text{RfD}}$$

The THQ stands for Target Hazard Quotient. EDI refers to the estimated daily intake of tested metals, measured in µg/kg bw/day. The reference doses (RfDs) established for non-carcinogenic effects were 1.0 µg/kg/day for Hg (USEPA, 2019)^18^, 0.1 µg/kg bw/day for Cd (USEPA, 2019)^18^, and 0.16 µg/kg bw/day for Pb (FDA, 2018)^19^.

The Total Target Hazard Quotients (TTHQs) were calculated by summing the THQs according to USEPA (2011)^20^ for each metal as follows:

$${\text{TTHQ}}={\text{TH}}{{\text{Q}}_{{\text{Hg}}}}+{\text{TH}}{{\text{Q}}_{{\text{Pb}}}}+{\text{TH}}{{\text{Q}}_{{\text{Cd}}}}$$

#### Cancer risk (CR) assessment

The CR can be evaluated to assess the creation of cancer over a lifespan from the consumption of metal-contaminated food according to the formula set by USEPA (2011)^20^.

$${\text{CR}}={\text{EDI}} \times {\text{CSF}}$$

Where CR refers to the cancer risk, EDI (mg/kg/day) is the estimated daily ingestion of each metal. The cancer slope factor (CSF) was 0.0085 and 0.38 mg/kg/day for lead and cadmium, respectively, while the CSF is not available for Hg^20^.

### Statistical analysis

The data obtained were subjected to the one-way analysis of variance to determine the differences in the heavy metal levels among the various tissue samples (beef, liver, and kidney) tested. Differences among the means of heavy metals were detected using Tukey’s Honestly Significant Difference (HSD) test (*P* < 0.05). The data were analyzed using GraphPad PRISM^®^ 9.1.2. (Graph Pad Software Incorporated, San Diego, USA).

## Results and discussion

### Heavy metal concentrations (mg/kg wet weight) in various tissues of imported frozen cattle samples

The mean concentrations of Hg in the examined bovine tissue samples ranged from 0.013 to 1.228 mg/kg in the meat, 0.001 to 1.190 mg/kg in the liver, and 0.001 to 0.790 mg/kg in the kidney, with mean levels of 0.312 ± 0.058, 0.273 ± 0.054, and 0.167 ± 0.04 mg/kg, respectively (Table 1). Beef samples had the highest mean concentrations of Hg compared to liver and kidney samples, with significant differences between the Hg in kidney and each of liver (P-value < 0.05) and muscle (P-value < 0.01) (Table 1). The Hg levels in the imported frozen bovine samples tested in this study are about 2.5 times higher than those reported in a previous study in Iraq, where the Hg concentration in imported frozen boneless beef of Brazilian origin tested in Iraq was 0.122 ± 0.006 mg/kg^21^; However much lower Hg levels of 0.003, 0.002, and 0.003 mg/kg were reported in bovine muscle, liver, and kidney, respectively, from Iran^22^. Additionally, a lower Hg level of 0.11 ± 0.02 mg/kg was detected in imported frozen bovine liver in Egypt^23^. Conversely, high Hg levels of 1.15 ± 0.27 and 1.18 ± 0.32 mg/kg were detected in bovine liver and kidney tested in Sharkia governorate, Egypt^24^.

The levels of Pb in the current study varied from 0.006 to 1.841 mg/kg in liver samples, 0.020 to 1.999 mg/kg in meat samples, and 0.003 to 2.050 mg/kg in kidney samples, with mean values of 0.763 ± 0.106, 0.684 ± 0.105, and 0.716 ± 0.119 mg/kg, respectively (Table 1). There was no significance among the mean levels of Pb in all the tested samples. However, high levels were detected in the liver and kidney samples. Such results are in accordance with the fact that heavy metals are accumulated more gradually in edible organs such as the liver and kidneys that have detoxification functions^25^.

The mean ± SE values of Pb determined in bovine tissue samples in this study were nearly similar to the contents of 0.69 ± 0.05 mg/kg in imported frozen bovine liver from Egypt^23^ and 0.672 ± 0.473 mg/kg detected in bovine liver collected from Slovakia^26^. Conversely, lower Pb levels of 0.221, 0.273, and 0.244 mg/kg were detected in bovine muscle, liver, and kidney, respectively, from Iran^22^. Furthermore, other lower Pb concentrations of 0.19 ± 0.02, 0.38 ± 0.08, and 0.32 ± 0.04 mg/kg were recorded in muscle, liver, and kidney from cattle carcasses in Egypt^24^. In contrast, higher Pb levels of 1.05 ± 0.12, 2.4 ± 0.19, and 1.33 ± 0.14 mg/kg were found in muscle, liver, and kidney from cattle carcasses in Egypt^27^.

**Table 1:** Comparison of the selected heavy metal concentrations (µg/g wet weight) in frozen imported bovine muscle, liver, and kidney samples with their international and National maximum permissible limit (MPL) (*n* = 105 for each tissue).

Heavy metal	Tissues	Minimum level	Maximum level	Mean ± SE	MPL (µg/g)	The number of samples exceeded the MPL	% of samples exceeded the MPL
Hg	Muscle Liver Kidney	0.013 0.001 0.001	1.228 1.190 0.790	0.312 ± 0.034^x^ 0.273 ± 0.031^x^ 0.167 ± 0.023^y^	0.05^a^ 0.05^a^ 0.05^a^	57 60 42	54.3% 57.1% 40%
Pb	Muscle Liver Kidney	0.020 0.006 0.003	1.999 1.841 2.050	0.684 ± 0.06^x^ 0.763 ± 0.061^x^ 0.716 ± 0.068^x^	0.1^b^ 0.5^b^ 0.5^b^	66 63 57	62.9% 60% 54.3%
Cd	Muscle Liver Kidney	0.007 0.010 0.011	0.100 0.390 0.211	0.030 ± 0.003^x^ 0.056 ± 0.008^y^ 0.073 ± 0.004^z^	0.05^c^ 0.5^c^ 1.0^c^	24 0 0	22.9% 0% 0%

^a^USDA (2023)^28^.

^b^EC (2015)^29^.

^c^EC (2014)^30^.

^x−z^Mean ± SE concentrations for each metal analyzed among the different organs with different superscript are significantly different (*P* < 0.05).

Cadmium levels in the current study were ranged found from 0.010 to 0.390 mg/kg in liver samples, 0.007 to 0.100 mg/kg in meat samples, and 0.011 to 0.211 mg/kg in kidney samples, with a higher mean concentration of 0.073 ± 0.014 mg/kg in the Kidney than in Liver (0.056 ± 0.007 mg/kg) and meat samples (0.030 ± 0.005 mg/kg), as noticed in Table 1. This finding claimed that the kidney is considered a vital organ that plays a role in metabolic processes and mineral storage, making it one of the most expressive indicators of any biological issue, and normally contains the highest levels of toxicants because they are responsible for excretion^22^.

The mean values of Cd detected in bovine tissue samples were comparable with those recorded in Egypt by Morshdy et al. (2018)^24^ at 0.03 ± 0.02, 0.06 ± 0.01 mg/kg in beef muscle and kidney samples, and by El-Ghareb et al. (2025)^23^ at 0.03 ± 0.001 mg/kg in imported frozen bovine liver. Similar to the Cd levels detected in the current study, Cd levels of 0.04 ± 0.01 mg/kg were reported in liver samples of female cattle carcasses from Nigeria^31^, as well as at concentrations of 0.05 ± 0.01, 0.17 ± 0.01 mg/kg, in liver and kidney samples from Bulgaria, respectively^32^. Nonetheless, lower Cd levels of 0.50 ± 0.00 µg/kg were reported in bovine liver samples collected from Nigeria^33^, as well as at a concentration of 0.023 ± 0.025 mg/kg in cattle meat samples collected from Bangladesh^34^. In contrast, a high Cd level of 4.31 mg/kg was found in beef samples collected from Iran^35^. Furthermore, high Cd levels of 0.079 ± 0.01, 0.11 ± 0.01, and 0.16 ± 0.01 mg/kg were detected in muscle, liver, and kidney from cattle carcasses in Egypt^27^.

The variations in heavy metal levels in this study and other studies worldwide are attributable to various factors, including feed sources, environmental contamination, animal age, and meat origin^36^. In addition, the different degrees of freezing, food packaging, and poor storage conditions available for the bovine sample storage can affect the levels of heavy metal residues^37^.

The summary of the analysis of variance (ANOVA) and Tukey HSD Test data for the heavy metals determined in the tissue analyzed is shown as supplementary data in Table S1.

### Heavy metals analyzed in bovine tissue samples tested in comparison to the legal limits

**Fig. 2:**
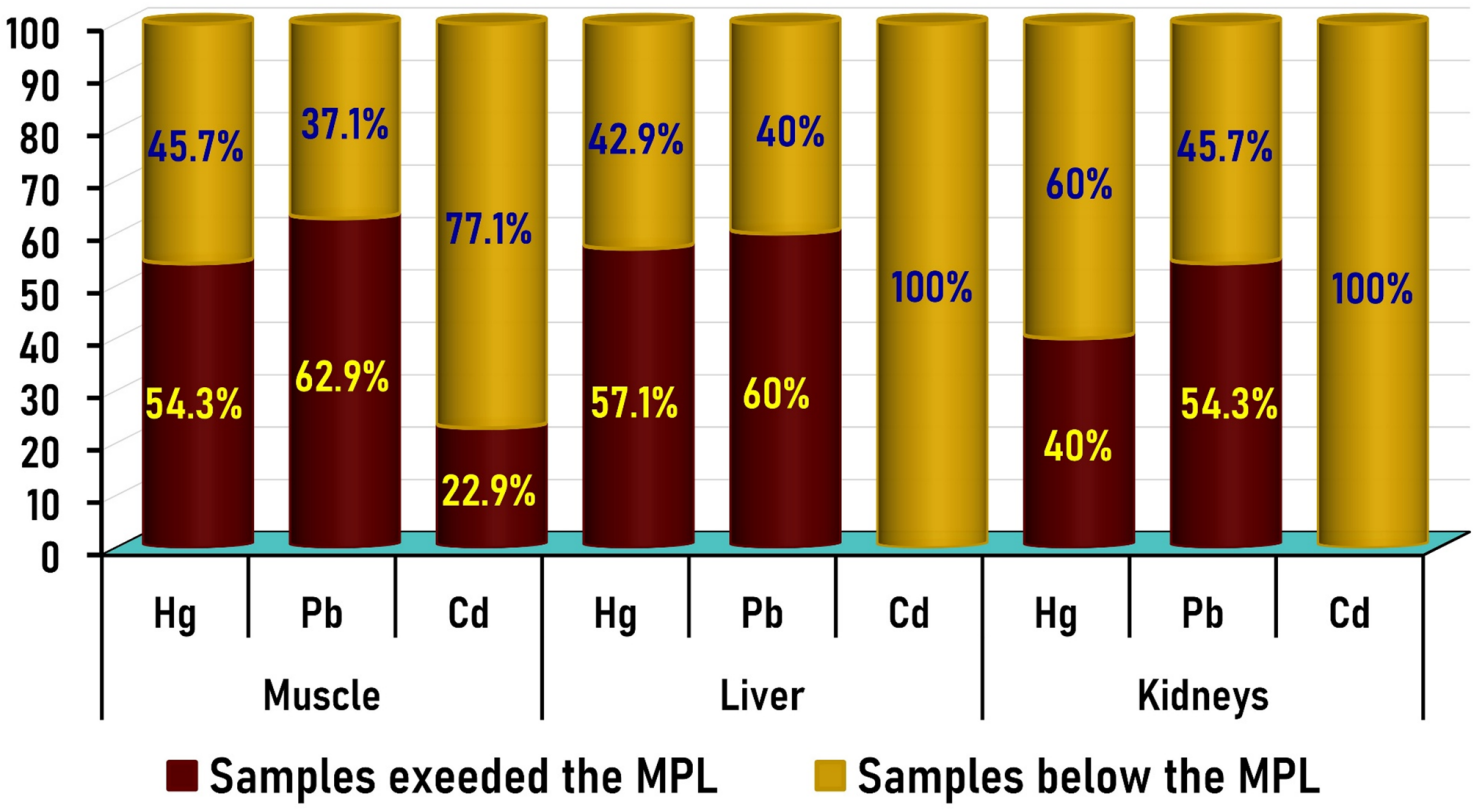
The percentage (%) of beef samples exceeded the MPLs versus that within the MPL for each metal in the three tested beef organs.

Our results indicated that 54.3% (57/105), 57.1% (60/105), and 40% (42/105) of the examined bovine meat, liver, and kidney samples, respectively, had Hg concentrations (Fig. 2) above the allowable limits set by the USDA (2023)^28^. Similarly, 75.3, 100, and 92.7% of bovine muscle, liver, and kidney samples tested previously in Egypt exceeded the allowable limits^24^. However, the result of the current study was in contrast with those reported by Hashemi in Iran^22^ and by Unguryanu et al. in Russia, who found that the Hg levels in all bovine muscle, liver, and kidney samples did not exceed the maximum recommended limits^38^.

Lead values in 62.9% (66/105), 60% (63/105), and 54.3% (57/105) of the examined bovine meat, liver, and kidney samples, respectively (Fig. 2), exceeded the MPL set by EC (2015)^29^. Conversely, 75%, 15.3%, and 13.9% of muscle, liver, and kidney collected from cattle carcasses, respectively, had Pb levels above the MPLs^22^. On the other hand, 100% of bovine tissue samples collected from Russia contained Pb levels above the maximum legal limits^38^. Furthermore, 81.3%, 88.7%, and 71.3% of meat, liver, and kidney samples collected from buffalo carcasses in Egypt, respectively, had Pb levels above the MPLs^27^.

In this study, 22.9% of the examined samples had Cd levels higher than MPLs, set by EC (2014)^30^, and recovered only from the beef samples, while the liver and kidney samples had Cd residue levels within the permissible limits (Fig. 2). Nearly similar results were recovered in bovines slaughtered in Iran, where the Cd concentrations in all liver and kidney samples did not exceed the maximum recommended limits, while 8.3% of bovine muscle samples were above the MPLs in Iran^22^. However, in another study, the Cd levels in all samples examined in Ethiopia, including beef muscle, were within permissible levels^39^. In contrast, Cd contents in 68% of bovine muscle samples collected from Egypt were above the maximum legal limits^27^.

### Health risks assessment associated with the intake of the tested tissues of imported frozen beef and liver

The process of identifying potential health effects of a specific toxicant on individuals through one or more exposure routes is called risk assessment. Concerns are rising regarding public health risks from heavy metal contamination in food. Heavy metals have been shown to impact cellular organelles and components, including the cell membrane, nuclei, lysosomes, enzymes, and mitochondria. Metal ions can bind to DNA and nuclear proteins, causing DNA damage that may lead to apoptosis or cancer^40^. The health risks associated with consuming bovine tissues, as examined in this study, were evaluated using the EDI, THQ, and TTHQ. The hazard health risk assessment was not conducted on kidney tissues because fewer samples exceeded the maximum permissible metal levels compared to meat and liver. Additionally, the per capita consumption of kidney is significantly lower than that of beef and liver.

#### Comparison of EDI of heavy metals detected in the examined beef and liver with their PTDI/BMDL for non-carcinogenic risk assessment

The calculated EDIs of Hg, Pb, and Cd from the consumption of examined bovine samples were lower than the PTDI/BMDL by 13.75%, 11.01%, and 0.366% in beef and 4.05%, 4.135%, and 0.3% in liver, respectively (Table 2). These findings suggested the absence of non-carcinogenic health risks from the consumption of imported frozen bovine meat and liver for Egyptian consumers. The results of the current study were in accordance with previous studies that estimated the EDI values for the Levels of Hg, Pb, and Cd contaminating meat and offal in Russia^38^, Egypt^41^, Nigeria^42^, and Bangladesh^43^. The current results suggest that the potential cancer risk from the consumption of such contaminated imported frozen food is very low or negligible. However, the periodic studies on the heavy metal residue levels in food are of great importance.

**Table 2:** Estimated daily intake (EDI) (µg /day/person) of detected heavy metals in the examined tissues of imported frozen cattle organs in comparison to their provisional tolerable daily intakes (PTDI) or their benchmark dose levels (BMDL).

Heavy metals	PTDI/BMDL µg/person/day	PTDI/BMDL µg/person/day	Mean conc of heavy metals (µg/kg)		Muscle		Liver	
			Muscle	Liver	EDI	% PTDI/BMDL	EDI	%PTDI/BMDL
Hg	0.23^a^	16.1	312	273	2.215	13.75	0.65	4.05
Pb	0.63^b^	44.1	684	763	4.856	11.01	1.82	4.135
Cd	0.83^c^	58.1	30	73	0.213	0.366	0.174	0.3

EDI = MC × IR according to Chien et al. (2002)^17^. Where EDI is the estimated daily intake (mg/kg/day), IR is the daily ingestion rate of imported cattle tissues by a 70-kg person in Egypt: 7.1 g (0.0071 kg) of muscle and 2.39 g (0.00239) of liver, calculated by the USDA (2022)^15^ and USDA (2020)^16^, respectively. MC is the metal concentration in bovine samples (mg/kg wet weight) (^a^JECFA (2010a)^44^; ^b^EFSA (2010)^45^; ^c^JECFA (2010b)^46^.

#### THQ and TTHQ of metals detected in the examined imported frozen beef and liver samples for non-carcinogenic risks assessment

THQ and TTHQ were applied to measure the health risks associated with beef and liver consumption. Consuming imported frozen beef contaminated with heavy metals would not have any negative non-carcinogenic health effects if the THQ or TTHQ is estimated to be less than 1; if the THQ or TTHQ is greater than 1, there would be a greater likelihood of non-carcinogenic chronic public health effects^47^. Although the THQ estimation methodology does not yield a quantitative estimate of the likelihood that an exposed population will have a reverse health consequence, it does provide an indicator of the risk level associated with pollutant exposure^48^.

The THQ and TTHQ values of metals tested in imported frozen beef and liver estimated in this study are shown in Table 3. The THQ index for Pb was 0.433 and 0.1625 in beef and liver, respectively. The THQ values for Cd in both beef and liver samples were 0.00304 and 0.00248, respectively. The THQ values for Hg were 0.316 and 0.0928 in beef and liver, respectively. In the current study, the total hazard (TTHQ) of Hg, Pb, and Cd was 0.752 from the consumption of contaminated beef, while the TTHQ value for Hg, Pb, and Cd collectively was 0.257 in liver (Table 3). The calculated THQ and TTHQ values for Pb and Cd were less than 1 in all examined beef and liver samples, reflecting that there were no potential risks to the Egyptian community associated with the intake of imported frozen bovine meat and liver contaminated with such metals. The THQ findings in this study are similar to the results of previous studies, where the THQ values of Pb and Cd were less than 1 from the consumption of beef in Iran^49^ and the analyzed bovine liver in Egypt^41^. Similarly, THQ values for Hg, Pb, and Cd were < 1.0 from the consumption of buffalo meat and liver in Egypt^27^. Contrastingly, previous studies revealed higher THQ values than the standard limits for Pb and Cd in beef samples examined in Uganda^50–52^. Furthermore, higher TTHQ values were recorded in beef liver samples collected from Bangladesh^43^ and Nigeria^53^, indicating the serious health effects associated with the consumption of such an organ.

Several studies stated that health risk assessments linked with toxic metals based on overall metal values in raw foods did not seem to be reasonable and resulted in overestimating the risk evaluation, since different preparation methods, which were not considered, such as soaking, washing, and heating foods before consumption, may decrease the heavy metal levels^13,54^.

**Table 3:** Target hazard quotient (THQ) and hazard index (HI; total target hazard quotient) of heavy metals due to the consumption of imported frozen muscle and liver of cattle in Egypt.

Heavy metals	RfD (µg/kg BW/day)	Muscle		Liver	
		EDI µg/kg bw/day	THQ	EDI µg/kg bw/day	THQ
Hg	0.1^a^	0.031	0.316	0.00928	0.0928
Pb	0.16^b^	0.069	0.433	0.0026	0.1625
Cd	1.0^a^	0.00304	0.00304	0.00248	0.00248
^c^HI or TTHQ			0.752		0.257

EDI: Estimated daily intake; BW: Body weight. THQ = EDI/RfD. RfD = the oral reference dose (µg/kg/day). RfD for Hg (MeHg) and Cd (USEPA, 2019)^a^^18^ RfD for Pb (FDA, 2018)^b^^19^. HI (Hazard Index) or TTHQ (Total Target Hazard Quotient) = THQ_Hg_ + THQ_As_ + THQ_Pb_ + THQ_Cd_ (USEPA, 2011)^c^^20^.

#### Cancer risks (CR) for assessment of carcinogenic risk from consumption of imported frozen beef and liver

The CR describes the potential cancer risks posed to an individual after ingesting the contaminated meat over a lifetime. Generally, the CR values above E-4 are regarded as unacceptable, below E-6 are negligible, and between E-6 and E-4 are acceptable. In the current study, the CR values of Cd and Pb detected in imported frozen bovine meat and liver were less than E-04 (Table 4). This suggested the lack of carcinogenic risks for Egyptian consumers from the consumption of such tested imported frozen bovine tissues; therefore, there are no reasons to be concerned about the continuous intake of such tested bovine tissues. these results are in accordance with those detected in Russia, where CR of Pb and Cd were less than E-04, indicating no risk associated with the consumption of meat and offal of cow^38^. Similar results were estimated in previous studies in Iran^49^ and in Bangladesh^43^, where the CR determined for Pb was within the acceptable value due to the consumption of cow meat and liver.

Additionally, in Egypt, the CR values for both Pb and Cd were also found to be within acceptable limits due to the consumption of buffalo meat and liver^24^ and imported frozen bovine liver^23^. In contrast, the CR of Cd was higher than the acceptable limits due to the intake of meat and liver of the cow^49^.

Although the present health risk assessment was based on raw tissue concentrations, it is important to consider that food processing and cooking (e.g., hamburger production, boiling, frying) may alter heavy metal levels, potentially reducing the actual exposure of consumers. Future studies should investigate the influence of these processing steps on heavy metal residues in beef-based products.

**Table 4:** The cancer risk (CR) associated with the consumption of imported frozen muscle and liver cattle by heavy metals in relation to the estimated daily intake (EDI) (µg/kg bw/day) and cancer slope factor (CSF) (µg/kg bw/day).

Heavy metals	CSF (mg/kg/day)*	Muscle		Liver	
		EDI (mg/kg bw/days)	CR	EDI (mg/kg bw/days)	CR
Mercury	–	–	–	–	–
Lead	0.0085	6.9E − 5	5.86E − 7	2.6E − 5	2.2E − 7
Cadmium	0.38	3.04E − 6	1.15E − 6	2.48E − 6	9.4E − 7

*USEPA (2011): “United States Environmental Protection Agency, 2011”^20^. bw: body weight.

## Conclusion

The current study focuses on the toxic heavy metal contents in imported frozen beef, liver, and kidney samples. The analysis illustrated that all beef and offal samples were contaminated with high levels of mercury, lead, and cadmium, with more than half of the samples recording levels that exceeded the MPLs for Hg and Pb in beef. The estimation of the human health risk was detected. Despite low calculated health risk indices (THQ, CR < 1), the frequent exceedance of legal limits (MPLs) indicates potential long-term hazards and the need for stronger monitoring of imports, including regulatory actions by local and international authorities, along with consumer awareness and veterinary interventions, should be taken to ensure the toxic metal concentrations in imported frozen bovine samples do not exceed the recommended limits.

## Supplementary Information

Below is the link to the electronic supplementary material.

Supplementary Material 1

## Data Availability

The data sets generated in this study are included within the article and its supplementary information files. Any additional information is available from the corresponding author upon request.
