# Supplementary material for: Health risk assessment of heavy metals in imported frozen bovine meat and organs marketed in Sohag, Egypt
Source: Sci Rep. 2025 Dec 14;15:43828. doi: 10.1038/s41598-025-29927-x (PMC12706026; doi:10.1038/s41598-025-29927-x)
Supplement: Supplementary file 1 — Supplementary Material 1 [file 41598_2025_29927_MOESM1_ESM.doc]

**Table S1.** ANOVA Summary for the heavy metals determined in the tissue analyzed (Independent Samples k=3)

| Metal | Source of Variation | **sum of Squares (SS)** | degrees of freedom (DF) | Mean Square (MS) | F-statistic | p-value | Tukey HSD Test |
| --- | --- | --- | --- | --- | --- | --- | --- |
| Hg | Between organs | 1.182839 | 2 | 0.591419 | 6.41 | 0.001870 | HSD[.05]=0.1; HSD[.01]=0.12  M1 vs M2 nonsignificant  M1 vs M3 P<.01  M2 vs M3 P<.05 |
|  | Within organs | 28.77832 | 312 | 0.092238 |
|  | Total |  | 314 |  |
| Pb | Between organs | 0.327911 | 2 | 0.163955 | 0.39 | 0.677386 | M1 vs M2 nonsignificant  M1 vs M3 nonsignificant  M2 vs M3 nonsignificant |
|  | Within organs | 129.967167 | 312 | 0.416561 |
|  | Total | 130.295078 | 314 |  |
| Cd | Between organs | 0.098112 | 2 | 0.049056 | 16.2 | <0.0001 | HSD[.05]=0.02; HSD[.01]=0.02  M1 vs M2 P<.01  M1 vs M3 P<.01  M2 vs M3 nonsignificant |
|  | Within organs | 0.944514 | 312 | 0.003027 |
|  | Total | 1.042626 | 314 |  |

M1 = mean of beef samples. M2 = mean of liver samples 2. M3 = mean of kidney samples.

SD = the absolute [unsigned] difference between any two sample means required for significance at the designated level.

HSD [0.05] for the .05 level; HSD [0.01] for the .01 level.
